# Dual Functionality of Papaya Leaf Extracts: Anti-Coronavirus Activity and Anti-Inflammation Mechanism

**DOI:** 10.3390/foods13203274

**Published:** 2024-10-16

**Authors:** Yujia Cao, Kah-Man Lai, Kuo-Chang Fu, Chien-Liang Kuo, Yee-Joo Tan, Liangli (Lucy) Yu, Dejian Huang

**Affiliations:** 1 Department of Food Science and Technology, National University of Singapore, Singapore 117542, Singapore; yujia.cao@u.nus.edu; 2 Infectious Diseases Translational Research Programme, Department of Microbiology and Immunology, Yong Loo Lin School of Medicine, National University of Singapore, Singapore 117545, Singapore; laikahman69@gmail.com (K.-M.L.); mictyj@nus.edu.sg (Y.-J.T.); 3 AgriGADA Biotech Pte Ltd., 8 Eu Tong Sen Street #17–82, The Central, Singapore 059818, Singapore; michael@vital-wellspring.com (K.-C.F.); cl.kcll@gmail.com (C.-L.K.); 4 Ph.D. Program for Aging, College of Medicine, China Medical University, Taichung 333, Taiwan; 5 Department of Nutrition and Food Science, University of Maryland, College Park, MD 20742, USA; lyu5@umd.edu; 6 Biomedical and Health Technology Platform, National University of Singapore (Suzhou) Research Institute, Suzhou 215123, China

**Keywords:** papaya leaves, anti-inflammation, anti-coronavirus, synergistic effect, COVID-19

## Abstract

Papaya leaves have been used as food and traditional herbs for the treatment of cancer, diabetes, asthma, and virus infections, but the active principle has not been understood. We hypothesized that the anti-inflammatory activity could be the predominant underlying principle. To test this, we extracted papaya leaf juice with different organic solvents and found that the ethyl acetate (EA) fraction showed the most outstanding anti-inflammatory activity by suppressing the production of nitric oxide (NO, IC_50_ = 24.94 ± 2.4 μg/mL) and the expression of pro-inflammatory enzymes, such as inducible nitric oxide synthase (iNOS) and cyclooxygenase (COX-2), and cytokines including interleukins (IL-1β and IL-6), and a tumor necrosis factor (TNF-α) in lipopolysaccharide (LPS)-induced RAW 264.7 cells. Transcriptomic analysis and Western blot results revealed its anti-inflammatory mechanisms were through the MAPK signaling pathway by inhibiting the phosphorylation of ERK1/2, JNKs, and p38 and the prevention of the cell surface expression of TLR4. Furthermore, we discovered that the EA fraction could inhibit the replication of alpha-coronavirus (HCoV-229E) and beta-coronavirus (HCoV-OC43 and SARS-CoV-2) and might be able to prevent cytokine storms caused by the coronavirus infection. From HPLC-QTOF-MS data, we found that the predominant phytochemicals that existed in the EA fraction were quercetin and kaempferol glycosides and carpaine. Counter-intuitively, further fractionation resulted in a loss of activity, suggesting that the synergistic effect of different components in the EA fraction contribute to the overall potent activity. Taken together, our results provide preliminary evidence for papaya leaf as a potential anti-inflammatory and anti-coronavirus agent, warranting further study for its use for human health promotion.

## 1. Introduction

Papaya (*Carica papaya*) is an herbaceous plant from the *Caricaceae* family. It originated from South America and is widely grown in tropical and subtropical regions [1]. While papaya fruits are nutritious and popular, papaya leaves receive much less attention and are discarded as agricultural waste [2]. Papaya leaves are consumed as a vegetable and traditional herb in many Asian countries [3]. Some evidence has suggested that the decoction of papaya leaves could ease cancer-related symptoms, and they were documented for the treatment of high blood pressure, diabetes, asthma, and virus infections [4,5,6]. However, there is no in-depth research on the scientific evidence or chemical principle responsible for such health-promotion use. It is reasonable to hypothesize that papaya leaves suppress inflammation, which is a common cause of these ailments [7]. A very limited study suggested that papaya leaves have anti-inflammatory activity. For example, the methanol extract of papaya leaves was reported to decrease the secretion of pro-inflammatory cytokines like tumor necrosis factor (TNF)-α, interleukin (IL)-1α, IL-1β, IL-6, and IL-8 in lipopolysaccharide (LPS)-stimulated human peripheral blood mononuclear cells (PBMCs) [8]. The anti-inflammatory activity of the ethanol extract of papaya leaves was further confirmed in vivo on carrageenan-induced paw edema, cotton pellet granuloma, and formaldehyde-induced arthritis models [9]. Further in-depth research is needed to understand the chemical components responsible for the activity and the related molecular biological mechanisms.

Although the highly contagious coronavirus disease 2019 (COVID-19) pandemic is over, the new variants are still emerging and making people sick. Therefore, new anti-coronavirus nutraceuticals are still in need. Severe acute respiratory syndrome coronavirus 2 (SARS-CoV-2) belongs to the family of *Coronaviridae*, a group of positive-sense and single-standard RNA viruses, including lethally transmitted viruses such as SARS-CoV and Middle East respiratory syndrome coronavirus (MERS-CoV) [10,11]. Apart from them, human coronavirus (HCoV)-OC43, HCoV-229E, HCoV-HKU1, and HCoV-NL63 are four known endemically transmitted HCoVs that usually lead to mild-to-moderate respiratory disease [12]. In terms of the molecular taxonomic hierarchy of coronaviruses, alpha-CoVs and beta-CoVs are our main concerns as they particularly infect mammals and humans [13]. SARS-CoV, SARS-CoV-2, MERS-CoV, HCoV-OC43, and HCoV-HKU1 belong to beta genera, yet HCoV-229E and HCoV-NL63 are alpha-CoVs [14].

It is witnessed that the evolution of HCoVs is rapid and unpredictable, resulting in variants with enhanced transmissibility or virulence [15]. Also, HCoVs are able to trigger the human immune system, leading to massive immune response such as a cytokine release with severe inflammation [16]. Several treatments such as immunomodulatory drugs, monoclonal antibodies, and antiviral agents have been applied to treat HCoVs-caused symptoms; however, their large-scale use is hindered due to their high cost, toxicity, and low efficacy [17]. In contrast, bioactive components extracted from natural plants with antiviral and anti-inflammatory properties, yet negligible cytotoxicity, gain more eligibility for the treatment of HCoVs infections [18]. Considering previous findings of the antiviral function of papaya leaves against SARS-CoV-2, dengue virus 2 (DENV-2), and chikungunya virus (CHIKV), along with the anti-inflammatory activity, it is hypothesized that papaya leaves may have potential in combating these HCoVs crises [19,20,21].

Hence, the objective of the present study was to elucidate the cellular anti-inflammatory signaling pathway of papaya leaves on LPS-stimulated RAW 264.7 cells and decode the potential bioactive compounds responsible for the anti-inflammation and antiviral activity against both alpha-CoVs (HCoV-229E) and beta-CoVs (HCoV-OC43 and SARS-CoV-2).

## 2. Materials and Methods

### 2.1. Materials

Dulbecco’s modified eagle medium (DMEM) with high-glucose, penicillin–streptomycin solution (100 IU/mL penicillin and 100 µg/mL streptomycin) and phosphate-buffered saline (PBS) were purchased from Hyclone Laboratories Inc. (Singapore). Fetal bovine serum (FBS), non-essential amino acid (NEAA), minimum essential medium (MEM), radio-immunoprecipitation assay (RIPA) buffer, BCA Protein Assay Kit, and Trizol were bought from Thermo Fisher Scientific Co., Ltd. (Singapore). The cell counting kit-8 (CCK-8) was purchased from Dojindo Molecular Technologies, Inc. (Kumamoto, Japan). The Griess reagent system and GoTaq^®^ qPCR Master Mix were purchased from Promega Pte Ltd. (Madison, Wisconsin, DC, USA). The iScript cDNA synthesis kit was supplied by Bio-Rad Laboratories Pte. Ltd. (Singapore). Lipopolysaccharide (*Escherichia coli* serotype 055: B5), paraformaldehyde (PFA), tetramethylbenzidin (TMB), formalin solution, crystal violet, Nirmatrelvir (PF-07321332), PhosSTOP™, HPLC-grade formic acid, and dimethyl sulfoxide (DMSO) were obtained from Sigma-Aldrich Co., Ltd. (Singapore). Antibodies used for Western blot, including iNOS, COX-2, TLR4, MAPKs family (p-ERK1/2. ERK1/2, p-p38, p38, p-JNK, and JNK), β-actin, and horseradish peroxidase (HRP)-labeled secondary antibody, were purchased from Cell Signaling Technology Inc. (Danvers, MA, USA). The primers were purchased from Integrated DNA Technologies Pte. Ltd. (Singapore). Anti-HCoV-OC43 nucleoprotein antibody clone 542-7D (Cat. MAB9013) was from Millipore Sigma. Monoclonal antibody 1A9 was generated using spike protein of SARS-CoV and shown to cross-react with spike protein of SARS-CoV-2, as described in the previous studies [22,23].

### 2.2. Sample Preparation

Fresh harvested papaya leaves (10 kg) were bought from Singaporean local supplier, Pasar Talk (Chuan Fresh Pte. Ltd., Singapore). Papaya leaves were washed with deionized (DI) water and juiced (4300 mL) using a juicer (TEFAL ZC6001 ultra slow juicer, Singapore). Then, 50 mL papaya leaf juice was freeze-dried by Buchi Standalone Freeze-Dryer (Buchi, Singapore). The dried solid was dissolved in DMSO to obtain a solution (100 mg/mL). The remaining papaya leaf juice was fractionated with hexane (He) (1:3, *v*/*v*) 5 times by liquid–liquid extraction method to afford He fraction. The left aqueous layer was extracted with diethyl ether (DE), ethyl acetate (EA), and n-butanol (Bu) in sequence (1:3 (*v*/*v*), 5 times). He, DE, EA, Bu, and water fractions were evaporated to dryness under reduced pressure after extraction, and the solid fractions were dissolved in DMSO for bioactivities tests. Acid–base extraction was employed to separate compounds in EA fraction based on their acid–base properties. Specifically, the EA fraction sample (1.5 g) was dissolved in dichloromethane (DCM, 10 mL) and the solution was extracted three times with sodium carbonate (2% (*w*/*v*), 30 mL), followed by three times with hydrochloric acid (1 M, 30 mL). The corresponding aqueous and organic layers were collected, respectively, and evaporated to dryness under reduced pressure. The residues were reconstituted with DMSO before bioassays were performed.

### 2.3. Cells and Viruses

Murine macrophage cell line RAW 264.7 (TIB-71), African green monkey kidney Vero E6 cells (CRL-1586), and human non-small cell lung cancer H1299 (CRL-5803) were obtained from the American Type Culture Collection (ATCC). RAW 264.7 cells were cultured with high-glucose DMEM supplemented with 10% FBS and 1% penicillin–streptomycin. Vero E6 and H1299 cell lines were cultured in DMEM with 10% FBS and 1% NEAA. All cell lines were maintained in the humidified incubator with 5% CO_2_ at 37 °C. HCoV-OC43 and HCoV-229E were obtained from ATCC. SARS-CoV-2 (hCoV-19/Singapore/NUS0001/2023, GISAID accession: EPI_ISL_19016298) was isolated from patient.

### 2.4. Cytotoxicity Test

RAW 264.7 cells, H1299 cells, and Vero E6 cells were seeded on a 96-well plate and incubated until cells formed a monolayer. After finishing the treatment with samples at indicated concentration, cell viability was measured using CCK-8 assay kit following manufacturer’s instructions. The absorbance at 450 nm was measured with the microplate reader (Tecan Infinite F200, Männedorf, Switzerland). Cell viability was calculated by the percentage of the sample group to the untreated control group.

### 2.5. Nitric Oxide Concentration Determination

RAW 264.7 cells were pretreated with samples for 1 h and then stimulated by LPS (100 ng/mL) for 23 h. After incubation, 50 µL culture supernatant of each well was mixed with Griess Reagent. The nitric oxide (NO) concentration was measured at the absorbance of 540 nm and quantified using nitrite standard curve with range from 1.56 to 100 μM. Normalized NO concentration was calculated by dividing NO concentration of samples by that of DMSO.

### 2.6. Western Blotting

RAW 264.7 cells were seeded on 6-well plate at the density of 1 × 10^6^ cells per well and incubated overnight. Cells were pre-incubated with samples for 1 h before the stimulation with LPS (100 ng/mL) for 23 h. Afterwards, cells were washed with PBS and lysed using the RIPA buffer containing phosphatase inhibitors (PhosSTOP™) at 4 °C for 15 min (Thermo Fisher, Singapore). Total protein concentrations of cell lysates were determined by the Pierce™ BCA Protein Assay Kit (Thermo Fisher Scientific, Inc.). Western blotting was conducted as previously reported [24]. The same membranes used for the detection of phosphorylated proteins were stripped, blocked, and re-tested with corresponding prototype protein antibody.

### 2.7. Real-Time Quantitative Polymerase Chain Reaction (qRT-PCR)

The total RNA of treated RAW 264.7 cells were isolated by Trizol reagent, and the concentrations were measured using BioDrop Duo (Bio Drop, UK). The synthesis of complementary DNA (cDNA) was proceeded by iScript^TM^ cDNA Synthesis Kit (Bio-Rad Laboratories, Inc.) on a Thermal Cycler (T100, Bio-Rad, Singapore). The qRT-PCR was carried out on an Applied Biosystems StepOnePlus^TM^ Real-Time PCR System using GoTaq^®^ qPCR Master Mix. Primer sequences used were listed in Table S1. C_T_ values were recorded automatically by StepOne^TM^ software (Version 2.2). The RNA expression levels of TNF-α, IL-6, and IL-1β were analyzed by $2^{-∆∆C_{T}}$ method with β-actin as the internal standard.

### 2.8. Transcriptome Analysis

Library construction and the RNA-sequencing were conducted by NovogeneAIT Genomics Singapore Pte Ltd (Singapore). Quantified libraries were established and sequenced on Illumina platforms’ effective library concentration. Clean reads were aligned to the reference genome using Hisat2 v2.0.5. Estimated gene expression levels were calculated by Fragments Per Kilobase of transcript sequence per Million base pairs sequenced (FPKM) based on the length of the gene and read counts mapped to genes. Differential expression analysis was performed using the DESeq2Rpackage (1.20.0). The statistical enrichment of differential expression genes (DEGs) was analyzed in Gene Ontology (GO) and Kyoto encyclopedia of genes and genomes (KEGG)’s pathways. Genes with an adjusted *p*-value ≤ 0.05 found by DESeq2 were considered as expressed differentially and reactome pathways with corrected *p*-value ≤ 0.05 were assigned as significantly enriched.

### 2.9. In-Cell Enzyme-Linked Immunosorbent Assay (ELISA)

Virus was pre-titrated in plaque-forming units (PFU) by plaque assays. H1299 cells and Vero E6 cells were seeded at the density of 1.0 × 10^4^ cells to 1.5 × 10^4^ cells per well and incubated overnight. Cells were infected with 50 µL mixture of indicated virus inoculum at the multiplicity of infection (MOI) of 0.025 to 0.1 and samples were diluted at the indicated concentration for 1 h at 37 °C. Then, 50 µL DMEM was added and cells were incubated for another 23 h before fixing with 4% PFA. Cells were permeabilized with 0.2% (*v*/*v*) Triton-X-100 (VWR Chemicals BDH, Radnor, PA, USA)/PBS and blocked with 10% (*v*/*v*) FBS/PBS. The primary anti-HCoV-OC43 nucleocapsid antibody and anti-SARS-CoV-2 1A9 antibody was added and incubated for 2 h at room temperature or overnight at 4 °C. Afterwards, HRP-labeled secondary antibody was incubated with cells for 1 h. The enzyme reaction was visualized using TMB substrate for 5 min and was stopped with stop solution (1 M sulfuric acid) before measuring the absorbance using Tecan Spark multimode microplate reader. The calculation was based on previous report [25].

(1) $$\mathrm{Neutralization}\;\mathrm{rate}=100\%-(A_{\mathrm{sample}}-A_{\mathrm{mock}})/(A_{\mathrm{DMSO}}-A_{\mathrm{mock}})\times 100\%$$

where A_mock_, A_DMSO_, and A_sample_ represent the absorbance of the non-infected, DMSO-treated, and sample-tested group at 450 nm, respectively.

### 2.10. Plaque Reduction Neutralization Test (PRNT)

H1299 cells and Vero E6 cells were seeded on a 12-well plate at a density of 1.5 × 10^5^ cells to 3.5 × 10^5^ cells per well and incubated in 10% FBS-supplemented DMEM overnight. The mixture of indicated virus inoculum (250 µL, 160 to 200 PFU/mL) and sample at indicated concentration was added to the respective wells. Cells were incubated for 1 h in an incubator at 37 °C in a 5% CO_2_ atmosphere with gentle shaking every 10 min to allow for virus adsorption. After adsorption, the inoculum was removed from the cells, and 2 mL of samples diluted in overlay containing 1% FBS in MEM and 1.2% Avicel (FMC BioPolymer, Philadelphia, PA, USA) was added and incubated with the cells. At the 3 days post-infection (DPI), the overlay was then removed from the cells, and cells were fixed by 10% formalin for 1 h before staining using 0.1% crystal violet. Finally, the crystal violet solution was discarded, and the cells were washed with reverse osmosis (RO) water and dried. The neutralization rate was calculated according to the work from Liu et al. [26].

Neutralization rate = 100% × [1 − (N_sample_/N_DMSO_)] (2)

where N_DMSO_ and N_sample_ represent the average number of plaques in the DMSO-treated and sample-tested group, respectively.

### 2.11. HPLC-QTOF-MS Characterization of Compounds in EA Fraction

EA fraction was diluted with HPLC-grade methanol and filtered through a 0.45 μm hydrophilic PTFE syringe filter. It was further identified by high-performance liquid chromatography quadrupole time-of-flight mass spectrometry (HPLC-QTOF-MS) (Waters Xevo G2-XS QTOF MS, Waters, Singapore). A HPLC column (Luna 5 µM C18(2) 100 Å, 250 × 10 mm; Phenomenex) was employed with two solvent systems: ultrapure H_2_O with 0.1% formic acid (A) and acetonitrile (B). The chromatographic method held a linear gradient from 95% A to 100% B for 30 min at a flow rate of 0.9 mL/min. Both positive and negative modes of the electrospray ionization (ESI) source were applied.

### 2.12. Data Analysis

Statistical analysis was carried out by GraphPad Prism (version 9.5.0). Multi-group data comparison was analyzed by one-way or two-way analysis of variance (ANOVA). A Duncan correction was selected for the post hoc test. The * *p* < 0.05, ** *p* < 0.01, *** *p* < 0.001, and **** *p* < 0.0001 were used to indicate the statistical significance, and * *p* < 0.05 was considered statistically significant.

## 3. Results and Discussion

### 3.1. Nitric Oxide Inhibitory Activity of Papaya Leaf Juice Extract

**Figure 1:**
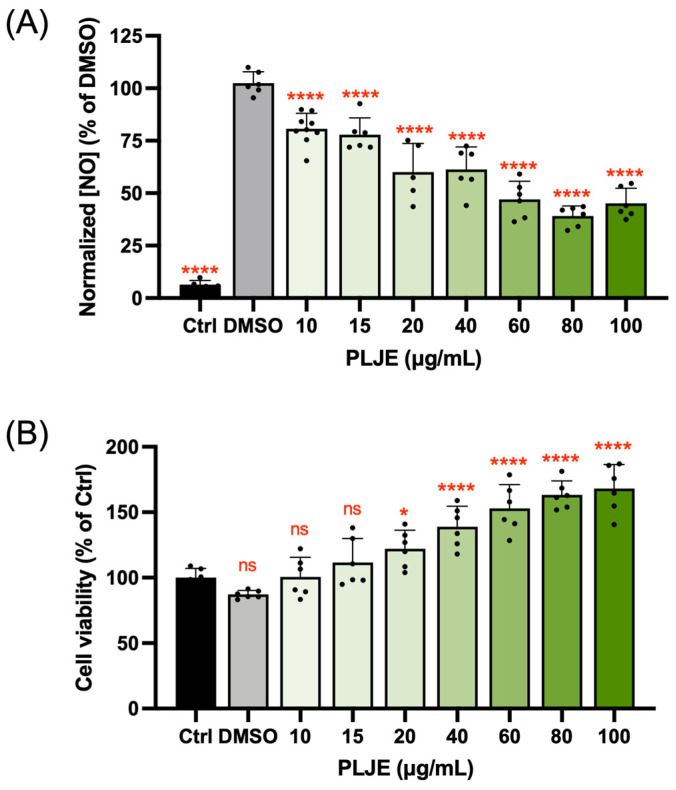
The inhibitory effects of papaya leaf juice extract (PLJE) on NO production in LPS-induced RAW 264.7 cells (**A**). Cytotoxicity of PLJE (**B**). Data points and bar represent arithmetic means ± SD. ns, not significant. * *p* < 0.05, **** *p* < 0.0001 compared to DMSO or control group.

The preliminary anti-inflammatory activity of papaya leaf juice extract (PLJE) was evaluated by determining the inhibition of nitric oxide (NO) production in LPS-induced RAW 264.7 cells. Figure 1A showed that PLJE could significantly suppress the production of NO with a dose–response trend. Remarkably, unlike some commercially prescribed drugs that show toxicity at a high concentration, a strong dose of PLJE boosted the cell viability of RAW 264.7 cells to about twice that of the control group, indicating the biosafety of using papaya leaves as an anti-inflammatory agent (Figure 1B).

### 3.2. NO Inhibitory Activity of Subfractions Extracted from Papaya Leaf Juice

**Figure 2:**
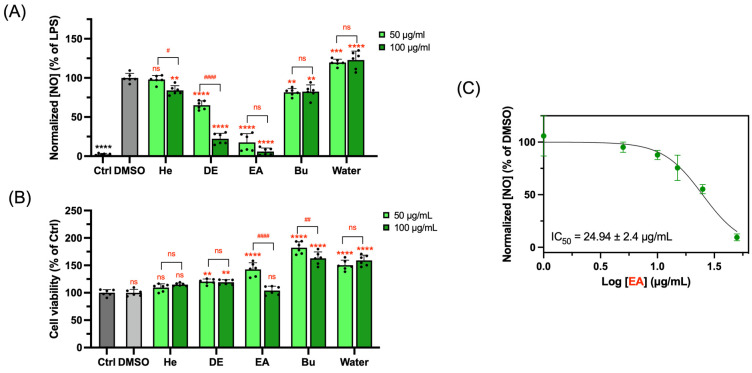
Suppressive effects of fractions extracted from PLJE on NO production in LPS-induced RAW 264.7 cells (**A**). Cell viability of RAW 264.7 cells treated with five fractions (**B**). IC_50_ of EA fraction on NO production in LPS-induced RAW 264.7 cells (**C**). Data points and bar represent arithmetic means ± SD. ns, not significant. ** *p* < 0.01, *** *p* < 0.001, and **** *p* < 0.0001 compared to DMSO group, ^#^ *p* < 0.05, ^##^ *p* < 0.01, and ^####^ *p* < 0.0001 compared within group.

To further investigate the anti-inflammatory effects of papaya leaves, fresh papaya leaf juice was fractionated by liquid–liquid extraction using solvents with an increased polarity into five sub-fractions. The water fraction accounted for the highest yield (80.54%), followed by the n-butanol and hexane fractions (9.00% and 7.92%, respectively). Diethyl ether (3.00%) and ethyl acetate (0.88%) ranked at the bottom. The EA fraction could be the most potentially effective fraction (Figure 2A) with the lowest normalized NO concentration at both tested concentrations. The Bu and DE fractions significantly suppressed the generation of NO as well, yet they could not reduce the NO production induced by LPS to the same level as EA. The half-maximal inhibitory concentration (IC_50_) of the EA fraction on NO production was 24.94 ± 2.4 μg/mL (Figure 2C). Importantly, all five fractions did not diminish the cell viability at 100 µg/mL (Figure 2B).

The phytochemicals that possibly existed in the papaya leaves were mainly alkaloids, phytate, saponin, flavonoids, tannins, and trace oxalate [27,28]. Among them, flavonoids and alkaloids were studies the most on their anti-inflammatory activity [29,30]. We attempted to investigate the regulatory potency of these two main categories of compounds in the EA fraction by separating according to their acid–base properties, but the results showed that the anti-inflammatory effects of all sub-fractions were inferior to the EA fraction (Figure S1). Based on these findings, our study mainly focused on the EA fraction with the greatest anti-inflammatory activity.

### 3.3. Suppressive Activity of EA Fraction on Pro-Inflammatory Protein and Cytokines

**Figure 3:**
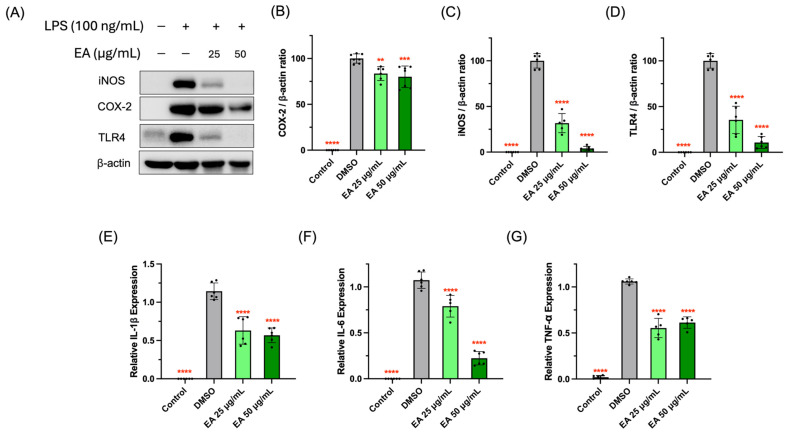
The inhibitory effects of EA fraction on inflammation-related protein expression in LPS-stimulated RAW 264.7 models (**A**). The expression levels of iNOS (**B**), COX-2 (**C**), and TLR4 (**D**) were determined by Western blot. The mRNA expression levels of IL-1β (**E**), IL-6 (**F**), and TNF-α (**G**) were tested by qRT-PCR. Data points and bar represent arithmetic means ± SD. ** *p* < 0.01, *** *p* < 0.001, and **** *p* < 0.0001 compared to DMSO group.

The overproduction of NO is likely due to iNOS, that usually participates in the pathogenesis of inflammatory diseases including atherosclerosis, rheumatoid arthritis, and diabetes [31]. The EA fraction could significantly block the expression of iNOS (Figure 3B), which suggests that the inhibitory effects of the EA fraction on NO production was mediated by the suppression of iNOS expression. COX-2, a common enzyme referring to prostaglandin synthesis, is the target of many commercially prescribed NSAIDs [32]. In our study, LPS stimulation remarkably elevated COX-2 expression and the EA fraction significantly attenuated the expression of the COX-2 protein (Figure 3A,C). In the interest of finding the function of the EA fraction on the cell surface ligand recognition of LPS and toll-like receptor 4 (TLR4), we evaluated the TLR4 levels as well. It was found that the EA fraction could significantly decrease the LPS-induced activation of TLR4 expression, protecting RAW 264.7 cells against inflammation (Figure 3C).

IL-1β, IL-6, and TNF-α are three major inflammatory cytokines with pro-inflammatory activities and participate in the interaction with a variety of pro-inflammatory mediators [33]. Our study found that the messenger ribonucleic acid (mRNA) expressions of IL-1β, IL-6, and TNF-α in RAW 264.7 cells significantly surged due to the stimulation of the LPSs. However, treatment with the EA fraction could attenuate their expression (Figure 3E–G), which was in agreement with the literature [8]. Overall, these results strongly suggested that the EA fraction inhibited the inflammatory activity in macrophage RAW 264.7 cells via the suppression of TLR4 activation, the protein expression of COX-2 and iNOS, and the gene expression of the pro-inflammatory cytokines IL-1β, IL-6, and TNF-α.

### 3.4. Transcriptome Analysis for the Potential Anti-Inflammatory Mechanisms of EA Fraction

**Figure 4:**
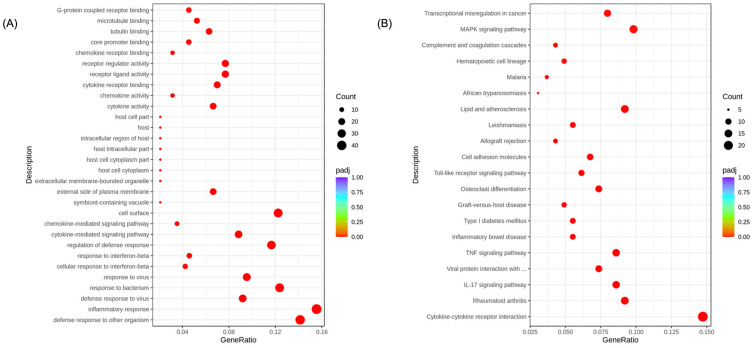
GO (**A**) and KEGG (**B**) enrichment scatter plots of EA (25 μg/mL) vs. DMSO group. GeneRatio is the ratio of the number of DEGs annotated to the GO or KEGG term to the total number of DEGs. The size of the dot represents the number of genes annotated to the terms.

Transcriptome analysis was employed to investigate the anti-inflammatory mechanisms of the EA fractions. The clean data of >12 Gb were collected for each sample from transcriptome sequencing with a Q20 value > 98% and Q30 value > 94% (Table S2). The squares of the Pearson correlation coefficient of the gene expression levels of all samples were all above 0.888 (Figure S2A). By comparing the RNA-sequencing results of the DMSO and control groups, it is revealed that LPS stimulation caused the alteration of 6024 DEGs in the gene transcriptome (Figure S2B). And, there were 160 genes in the DMSO group that were upregulated and 203 genes that were downregulated by the EA fraction, generating a volcano map (Figure S2C). The cluster analysis on the FPKM values of the genes showed that there was a significant difference between the triplicate runs of the DMSO groups and EA groups (Figure S3). The DEGs were subjected to a GO enrichment analysis for functional classification and the significantly enriched GO terms are plotted in Figure 4A. Notably, the inflammatory responses (GO:0006954), defense response to other organisms (GO:0098542), and defense to bacterium (GO:0009617) terms within the biological processes category ranked top, emphasizing the effect of the EA fraction on immunoregulation and defense for foreign invasions. In addition to the biological processes, the significant enriched cell surface (GO:0009986) term from the cellular component category highlighted the function of EA to impede the interaction of TLR4 and LPS on the RAW 264.7 cell surface.

To understand the related pathways associated with identified DEGs, KEGG enrichment was performed and the most significant 20 KEGG pathways are mapped in Figure 4B. Among these pathways, the cytokine–cytokine receptor (mmu04060) showed the highest GeneRatio of 14.72% and was associated with 24 DEGs (Figure 4B). Markedly, the MAPK signaling pathway (mmu04010), that had been reported as one of the most crucial pathways for LPS-induced inflammation, ranked second among these pathways (GeneRatio: 9.82%, Figure 4B). In addition, the IL-17 signaling pathway (mmu04657) and TNF signaling pathway (mmu04668) were found to be significantly changed with a GeneRatio of 8.59%. Remarkably, the EA fraction treatment significantly contributed to the regulation of inflammatory disorders such as rheumatoid arthritis (mmu05323), transcriptional mis-regulation in cancer (mmu05202), and inflammatory bowel disease (mmu05321) as well. It is also worth mentioning that there were 10 DEGs participating in the cell surface TLR signaling pathway (mmu04620), which was consistent with our Western blot and GO results (Figure 3D and Figure 4A).

### 3.5. Effects of EA Fraction on Suppressing MAPK Signaling Pathway

**Figure 5:**
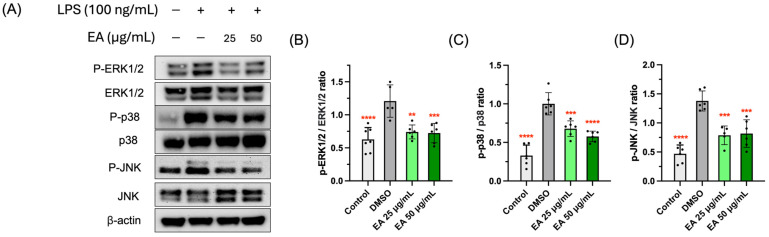
Effects of EA fraction on MAPK pathway in LPS-induced RAW 264.7 cell (**A**). Suppressive effects of EA fraction on the LPS-induced phosphorylation ratio of ERK1/2 (**B**), JNK (**C**), and p38 (**D**). Data points and bar represent arithmetic means ± SD. ** *p* < 0.01, *** *p* < 0.001, and **** *p* < 0.0001 compared to DMSO group.

**Figure 6:**
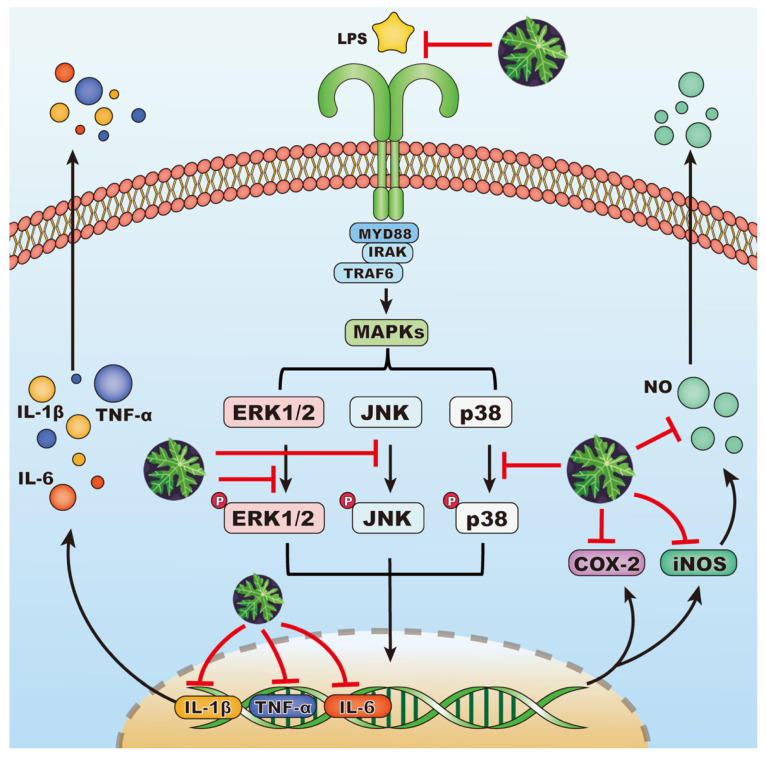
Schematic diagram of potential contribution of papaya leaves in LPS-induced signaling pathways.

MAPKs, a group of serine/threonine protein kinases, are involved in the conversion of extracellular stimulants to a wide spectrum of biological activities in macrophages [34,35]. Delving into the study of the molecular mechanism underlying anti-inflammatory mechanisms of the EA fraction on LPS-stimulated cells, we examined its effect on the expression of three key kinases from MAPK cascades: extracellular signal-regulated kinases 1/2 (ERK1/2), Jun amino-terminal kinases (JNK), and p38 mitogen-activated protein kinases (p38). LPSs brought about the significant phosphorylation of MAPKs (Figure 5), while the EA fraction could significantly suppress the phosphorylated MAPKs expression levels and reduce the phosphorylation ratio. This indicated that the EA fraction blocked ERK1/2, JNK, and p38 phosphorylation in the MAPK pathway, thereby inhibiting the LPS-induced inflammatory response in RAW 264.7 macrophages. Taken together, the inhibitory mechanism of the EA fraction against LPS-induced inflammation is demonstrated in Figure 6.

### 3.6. Anti-Coronavirus Activity of EA Fraction

To investigate the anti-coronavirus potential of papaya leaves, we firstly utilized a rapid in-cell ELISA-based neutralization assay to screen the effects of five fractions on the HCoV-OC43-infected H1299 cells. It was found that the EA fraction not only had excellent anti-inflammatory activity, but also an incomparable ability to decrease the replication of HCoV-OC43 (Figure S4A). Moreover, the subfractions could only neutralize about 50% of HCoV-OC43 at the concentration of 1.25 μg/mL or higher (Figure S4B), suggesting some synergistic effects among the components in the EA fraction. Therefore, the EA fraction is further studied for its efficacy. Nirmatrelvir (PF-07321332) is an oral protease inhibitor approved by the Food and Drug Administration (FDA) to act against the 3C-like proteinase protein (3CLPro/Mpro) of SARS-CoV-2 and has also been proved as an inhibitor on HCoV-OC43, HCoV-NL63, and HCoV-229E [36,37]. Thus, we included nirmatrelvir as a reference standard to evaluate the anti-coronavirus efficiency of the EA fraction and then determined the IC_50_ and selectivity index (SI) against beta-coronaviruses, which are shown in Table 1. Both the EA fraction and nirmatrelvir manifested anti-HCoV-OC43 activity with SI which was greater than 600. Remarkably, the EA fraction exhibited a higher anti-SARS-CoV-2 capability with a lower IC_50_ (0.1853 ± 0.0184 μg/mL) compared to nirmatrelvir (Table 1). The potent activity of the EA fraction against two beta-HCoVs support the utility of papaya leaves as an anti-beta-CoVs agent (Table 1).

**Figure 7:**
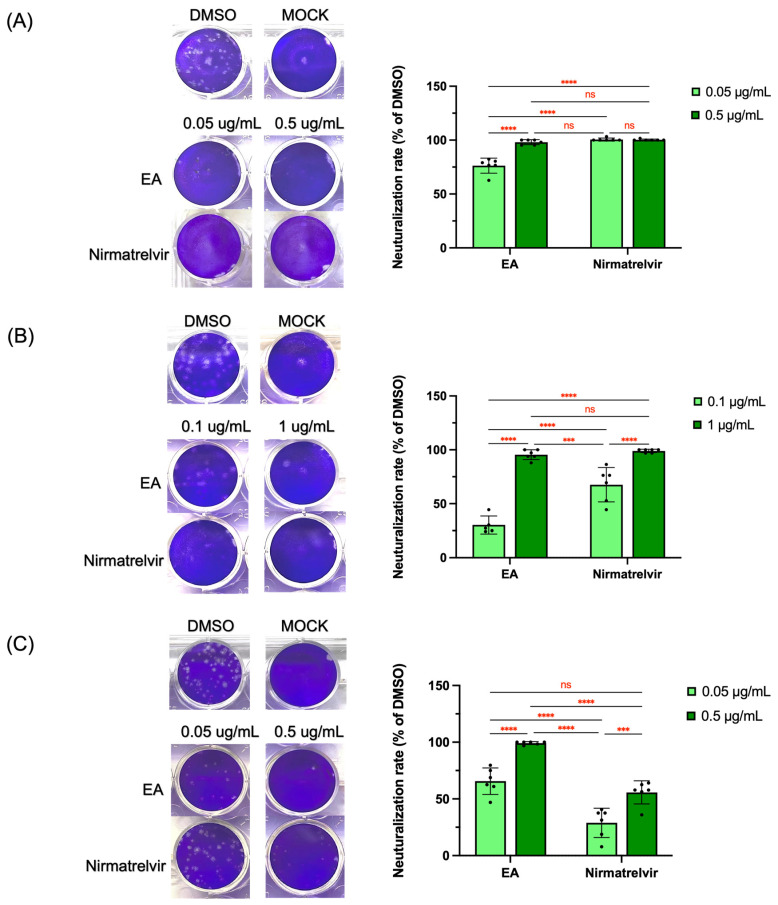
Plaque reduction neutralization tests (PRNT) of EA fraction and nirmatrelvir against infectious HCoVs-OC43 (**A**), HCoV-229E (**B**), and SARS-CoV-2 (**C**). Data points and bar represent arithmetic shown which are the mean ± SD of at least two independent tests performed. ns, not significant. *** *p* < 0.001, and **** *p* < 0.0001 compared between indicated groups.

The ELISA assay suffers from vulnerable antibody stability and inadequate inhibition and is prone to false-positive and -negative results. Therefore, PRNT, a more sensitive method, was performed to further confirm the ability of the EA fraction on the blockage of the viral replication of both beta- and alpha-CoVs. In Figure 7, the positive control (DMSO) group with a virus infection showed numerous plaques by virus-induced cell lysis. In contrast, the treatment groups by the EA fractions of papaya leaves or nirmatrelvir at tested concentrations significantly reduced the replication of HCoV-OC43, HCoV-229E, and SARS-CoV-2 (Figure 7A–C). Although viral replication was significantly inhibited by the EA fraction at lower concentrations (0.05 μg/mL for HCoV-OC43 and 0.1 μg/mL for HCoV-229E), its potency was not comparable to nirmatrelvir (Figure 7A,B). Unexpectedly, the anti-SARS-CoV-2 activity of the EA fraction was stronger than nirmatrelvir at the same concentration (Figure 7C). Moreover, the effect of the EA fraction at 0.05 μg/mL was statistically identical to nirmatrelvir at 0.5 μg/mL (Figure 7C). Our findings from PRNT were consistent with the results of the in-cell ELISA results that showed that the EA fraction had a lower IC_50_ against SARS-CoV-2, but a higher IC_50_ against HCoV-OC43 compared to nirmatrelvir (Table 1).

Clinical trials indicated that the immunopathogenesis of the SARS-CoV-2 infection was related to the hyperinflammatory state referred as a cytokine storm, which is marked by the elevation of IL-6, IL-1β, and TNF-α secreted from macrophages [38]. We found that the EA fraction could suppress the mRNA expression of the pro-inflammatory cytokines IL-1β, IL-6, and TNF-α in LPS-activated RAW 264.7, which suggests that the EA fraction could potentially prevent the inflammation storm triggered by SARS-CoV-2 infection.

### 3.7. HPLC-QTOF-MS Analysis of Phytochemicals in EA Fraction

The elemental composition information of potential compounds in the EA fraction was identified by HPLC-QTOF-MS, referring to the retention time (RT), their precursor ions, and fragment ions. The elution profile and total ion chromatogram is shown in Figure S5. Several compounds were tentatively identified as potential phytochemicals in Table 2. We found that the principal compounds in the EA fraction were flavonoids, specifically quercetin glycosides (manghaslin and rutin) and kaempferol glycosides such as clitorin and nicotiflorin, and one alkaloid called caprine that was unique to papaya leaves. Most of these identified compounds had been reported as present in papaya leaves before [19,39,40].

Rutin was proved to effectively prevent the expression of TLR4 and inhibit phosphorylation reactions in the NF-κB pathway in LPS-induced RAW 264.7 cells [41]. However, in the study of Shen et al., the rutin did not show inhibitory effects on 50 ng/mL LPS-induced NO production, iNOS, or COX-2 expression even at 80 μM [42]. In this controversial case, we tested the anti-inflammatory effects of rutin under the same circumstance of the EA fraction. To our surprise, rutin itself did not show an inhibitory effect on the production of NO even at 100 μg/mL (Figure S6A). There are few reports of the anti-inflammatory activity of other identified compounds except for nicotiflorin, which only exerted anti-inflammatory activity at a very high concentration of about 1 mg/mL [43].

Based on the molecular docking results from a previous study, manghaslin, rutin, clitorin, nicotiflorin, kaempferol-3-O-neohesperidoside, kaempferol 3-O-β-D-glucopyranoside, and carpaine were able to be docked into multitargets of SARS-CoV-2 such as Mpro, RNA-dependent RNA polymerase (RdRp), papain-like protease (PLpro), and the receptor-binding domain (RBD)-spike protein [19,44,45]. Importantly, rutin was the only compound reported for the anti-HCoV-229E in Huh-7 cells by reducing the development of the viral cytopathic effect (CPE) [46]. Nevertheless, in our study, rutin failed to neutralize HCoV-OC43 in H1299 cells at 10 μg/mL, which was a concentration about 100 times higher than the IC_50_ of the EA fraction (Figure S6B). Thus, it was speculated that combining compounds might have synergistic effects via different molecular targets and pathways, giving the EA fraction high potency as an anti-inflammatory and anti-coronavirus agent. The synergistic therapeutic functions of herbal extracts have been frequently reported. The combination therapy of alkaloids and flavonoids can enhance the activities of glycemic control and the inhibition of glycinergic transmission, contributing to preventing diabetes and hyperekplexia [47,48]. Furthermore, combinations of different flavonoids were observed to have synergistic effects in antioxidant, anti-diabetic, and anticancer actions [49,50,51]. However, the mechanisms of action of multiple compounds and their interaction are still not clear.

Additionally, the anti-coronavirus effects of the edible plant extracts were demonstrated before, suggesting an alternative therapy of using nutraceuticals and functional foods to control the development of contagious COVID-19 diseases [52]. Our study provides evidence for the potential utilization of the EA fraction extracted from papaya leaves to inhibit the replication of SARS-CoV-2 and relieve inflammation, thereby diminishing the risk of SARS-CoV-2 infection and alleviating COVID-19 symptoms. However, the dual functionality of papaya leaves and their mechanisms in more complicated physiological environments, such as animals and humans, are required to be further investigated. Also, the kinetic processes of the absorption, distribution, metabolism, and excretion (ADME) of papaya leaves in animals and humans need to be determined.

## 4. Conclusions

In conclusion, the EA fraction extracted from papaya leaf juice showed the most powerful anti-inflammatory and anti-coronavirus activity. We found it exerted anti-inflammatory effects in LPS-stimulated RAW 264.7 cells predominantly through the MAPK signaling pathway by blocking the phosphorylation of ERK1/2, JNK, and p38 and hindering the activation of TLR4, therefore suppressing the expression of pro-inflammatory enzymes and cytokines. The anti-coronavirus effect of the EA fraction was discovered using intracellular ELISA and PRNT against HCoV-OC43 and SARS-CoV-2 belonging to the beta-coronaviruses and the alpha-coronavirus HCoV-229E. The main phytochemicals present in the EA fraction analyzed by HPLC-QTOF-MS were flavonoids, especially quercetin and kaempferol glycosides, and caparine. Surprisingly, we found that a single compound in the EA fraction could not exert equally powerful bioactivity as the entire fraction, thus the synergistic effect of different components which exist in the EA fraction are responsible for its potent activity. Our results highlight the potential pharmaceutical utility of papaya leaves for the treatment of inflammation and coronavirus infections.

## Figures and Tables

**Table 1:** Antiviral activity and toxicity of EA fraction and nirmatrelvir against beta-CoVs.

Samples (μg/mL)	IC_50_ (μg/mL)		CC_50_ (μg/mL)		SI	
	HCoV-OC43 on H1299 (MOI = 0.1)	SARS-CoV-2 on Vero E6 (MOI = 0.025)	H1299	Vero E6	HCoV-OC43 on H1299 (MOI = 0.1)	SARS-CoV-2 on Vero E6 (MOI = 0.025)
EA	0.1247 ± 0.0078	0.1853 ± 0.0184	294.7 ± 22.7	>100	2363	>539.7
Nirmatrelvir	0.03873 ± 0.00316	0.3416 ± 0.0477	>25	>25	>645.5	>73.19

Selectivity index (SI) was calculated as the ratio of the CC_50_ against its IC_50_. Data shown are the mean ± SD of at least two independent tests performed.

**Table 2:** Phytochemicals identified (tentative) in EA fraction of papaya leaves.

RT (min)	Main Observed *m*/*z*	Quantification Ions	MS/MS Fragmentation (*m*/*z*)	Possible Formula	Experimental Mass	Error (ppm)	Suggested Compounds
9.12	479.3845	[M + H]^+^	337.19102; 217.13337; 121.06514	C_28_H_50_N_2_O_4_	478.37706	0.4	Carpaine
9.28	757.2209	[M + H]^+^	303.05058; 121.06528	C_33_H_40_O_20_	756.21129	3.0	Manghaslin
9.29	755.2084	[M − H]^−^	300.02432; 178.99403	C_33_H_40_O_20_	756.21129	6.5	Manghaslin
9.79	741.2257	[M + H]^+^	287.05555; 240.19609; 153.01867	C_33_H_40_O_19_	740.21638	2.8	Clitorin
9.80	739.2131	[M − H]^−^	285.03526; 284.02927; 178.99423	C_33_H_40_O_19_	740.21638	5.5	Clitorin
10.41	611.1619	[M + H]^+^	303.05017; 229.05013; 153.01869	C_27_H_30_O_16_	610.15338	2.8	Rutin
10.41	609.1478	[M − H]^−^	301.03157; 300.02455; 271.02135	C_27_H_30_O_16_	610.15338	2.8	Rutin
10.67	595.1664	[M + H]^+^	287.05510; 240.19552; 153.01845; 91.05425	C_27_H_30_O_15_	594.15847	1.2	Kaempferol-3-O-neohesperidoside
10.67	593.1528	[M − H]^−^	285.03523; 284.02950; 225.02632	C_27_H_30_O_15_	594.15847	2.8	Kaempferol-3-O-neohesperidoside
11.12	595.1656	[M + H]^+^	287.05411; 153.01810	C_27_H_30_O_15_	594.15847	−0.2	Nicotiflorin
11.12	593.1519	[M − H]^−^	285.03652; 284.02909 255.02596	C_27_H_30_O_15_	594.15847	1.2	Nicotiflorin
11.32	495.3773	[M + H]^+^	240.19491; 222.18426	C_26_H_48_N_5_O_4_	474.37063	−1.3	Unknown
11.82	449.1059	[M + H]^+^	287.05374; 153.01770	C_21_H_20_O_11_	448.10056	−4.4	Kaempferol-3-O-β-D-glucopyranoside
11.82	447.0911	[M − H]^−^	285.03526; 284.02885; 255.02570; 227.03053; 195.06156	C_21_H_20_O_11_	448.10056	−4.9	Kaempferol-3-O-β-D-glucopyranoside
12.02	428.2976	[M + H]^+^	287.05333; 240.19400; 222.18360	C_18_H_41_N_3_O_8_	427.28937	2.3	Unknown
12.02	635.1631	[M − H]^−^	301.03095; 284.02913; 119.04588	C_29_H_32_O_6_	636.16903	2.1	Unknown

## Data Availability

The original contributions presented in the study are included in the article/Supplementary Material, further inquiries can be directed to the corresponding author.

## Supplementary Materials

The following are available online at https://www.mdpi.com/article/10.3390/foods13203274/s1. Table S1. Primer sequences for RT-qPCR. Table S2. Quality of RNA-Sequencing results. Figure S1. Anti-inflammatory activity measured by suppressing nitric oxide production of subfractions from EA fraction on LPS-induced RAW 264.7 cells. Figure S2. Inter-sample correlation heat map (A). R2 represents square of Pearson correlation coefficient. The volcano plots analysis of differential expression genes (DEGs) between control and DMSO group (B) and DEGs between EA (25 μg/mL) and DMSO group (C). Threshold: padj < 0.05, |log2FoldChange| > 0. Figure S3. Differential expression gene clustering heatmap of EA- and DMSO-treated groups. Figure S4. Screening results of subfractions extracted from papaya leaves juice (A) and from EA fraction (B) on HCoV-OC43-infected H1299 cells using in-cell ELISA. Figure S5. HPLC profile (A) and total ion chromatogram in negative mode (B) and positive mode (C) of EA fraction. Figure S6. Inhibitory effects of rutin on NO production in LPS-induced RAW 264.7 cells (A) and its function in neutralizing HCoV-OC43 on infected H1299 cells (B).
