# Supplementary material for: Dual Functionality of Papaya Leaf Extracts: Anti-Coronavirus Activity and Anti-Inflammation Mechanism
Source: Foods. 2024 Oct 16;13(20):3274. doi: 10.3390/foods13203274 (PMC11506937; doi:10.3390/foods13203274)
Supplement: Supplementary file 1 [file foods-13-03274-s001.zip › foods-3263880-supplementary.pdf]

Supporting information

Dual Functionality of Papaya Leaf Extracts: Anti-Coronavirus Activity and Anti-Inflammation Mechanism

Yujia Cao [1], Kah-Man Lai [2], Kuo-Chang Fu [3], Chien-Liang Kuo [3,4], Yee-Joo Tan [2], Liangli (Lucy) Yu [5] and Dejian Huang [1,6,*]

[1] Department of Food Science and Technology, National University of Singapore, Singapore 117542, Singapore
[2] Infectious Diseases Translational Research Programme, Department of Microbiology and Immunology, Yong Loo Lin School of Medicine, National University of Singapore, Singapore 117545, Singapore
[3] AgriGADA Biotech Pte Ltd., 8 Eu Tong Sen Street #17–82, The Central, Singapore 059818, Singapore
[4] Ph.D. Program for Aging, College of Medicine, China Medical University, Taichung 333, Taiwan
[5] Department of Nutrition and Food Science, University of Maryland, College Park, MD 20742, USA
[6] Biomedical and Health Technology Platform, National University of Singapore (Suzhou) Research Institute, Suzhou 215123, China
* Correspondence: dejian@nus.edu.sg; Tel.: +65-82836618

Table S1. Primer sequences for RT-qPCR.

Gene	Forward strand	Reverse strand
β-actin	5′-CCACAGCTGAGAGGGAAATC-3′	5′-AAGGAAGGCTGGAAAAGAGC-3′
IL-1β	5′-GGGCCTCAAAGGAAAGAATC-3′	5′- TACCAGTTGGGGAACTCTGC-3′
IL-6	5′-AGTTGC CTTCTTGGGACTGA-3′	5′-CAGAATGCCATTGCACAAC-3′
TNF-α	5′-AGCCCCCAGTCTGTATCCTT-3′	5′-CATTCGAGGCTCCAGTGAAT-3′

Table S2. Quality of RNA-Sequencing results.

**Sample**	**Raw reads**	**Raw bases**	**Clean reads**	**Clean bases**	**Error rate (%)**	**Q20 (%)**	**Q30 (%)**	**GC (%)**
Ctrl_1	93864536	14.08G	92501342	13.88G	0.02	98.33	95.08	49.73
Ctrl_2	97882494	14.68G	96339450	14.45G	0.02	98.01	94.28	49.83
Ctrl_3	95843816	14.38G	94374866	14.16G	0.02	98.39	95.16	49.73
DMSO_1	103522778	15.53G	101241782	15.19G	0.02	98.21	94.87	49.63
DMSO_2	91795980	13.77G	90355588	13.55G	0.02	98.26	94.83	50.12
DMSO_3	88157656	13.22G	86361746	12.95G	0.02	98.35	95.1	49.96
EA_1	86549196	12.98G	83872508	12.58G	0.02	98.3	94.84	50.78
EA_2	93648312	14.05G	90522042	13.58G	0.02	98.17	94.59	50.92
EA_3	93701614	14.06G	91092302	13.66G	0.02	98.34	94.97	50.57

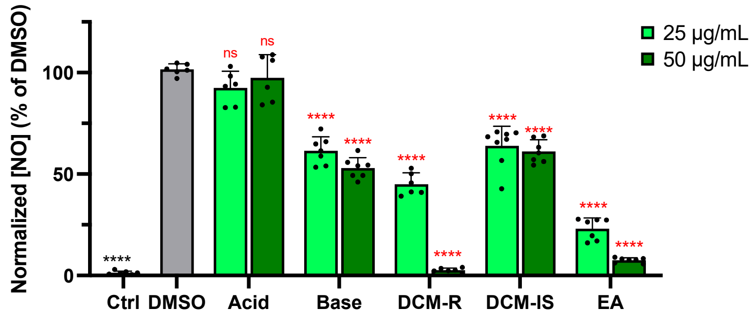

Figure. S1. Anti-inflammatory activity measured by suppressing nitric oxide production of subfractions from EA fraction on LPS-induced RAW 264.7 cells. Data points and bar represent arithmetic means ± SD. $^{*}P < 0.05$, $^{**}P < 0.01$, $^{***}P < 0.001$, $^{****}P < 0.0001$ as compared to DMSO only treated LPS-induced group. DCM-R: DCM residue; DCM-IS: DCM insoluble fraction.

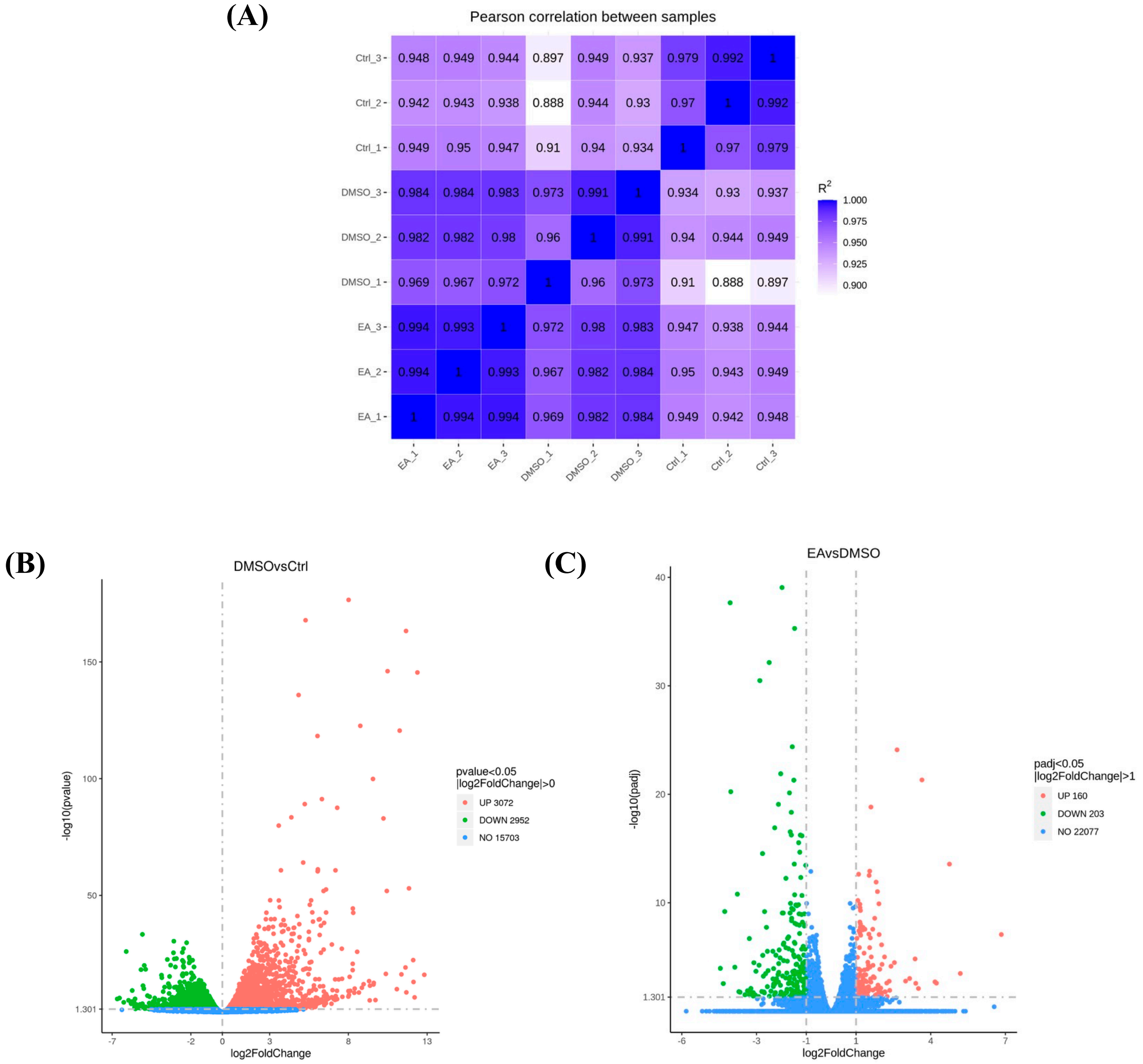

Figure. S2. Inter-sample correlation heat map (**A**). R^2 represents square of Pearson correlation coefficient. The volcano plots analysis of differential expression genes (DEGs) between control and DMSO group (**B)** and DEGs between EA (25 μg/mL) and DMSO group (**C**). Threshold: padj<0.05, |log2FoldChange|>0.

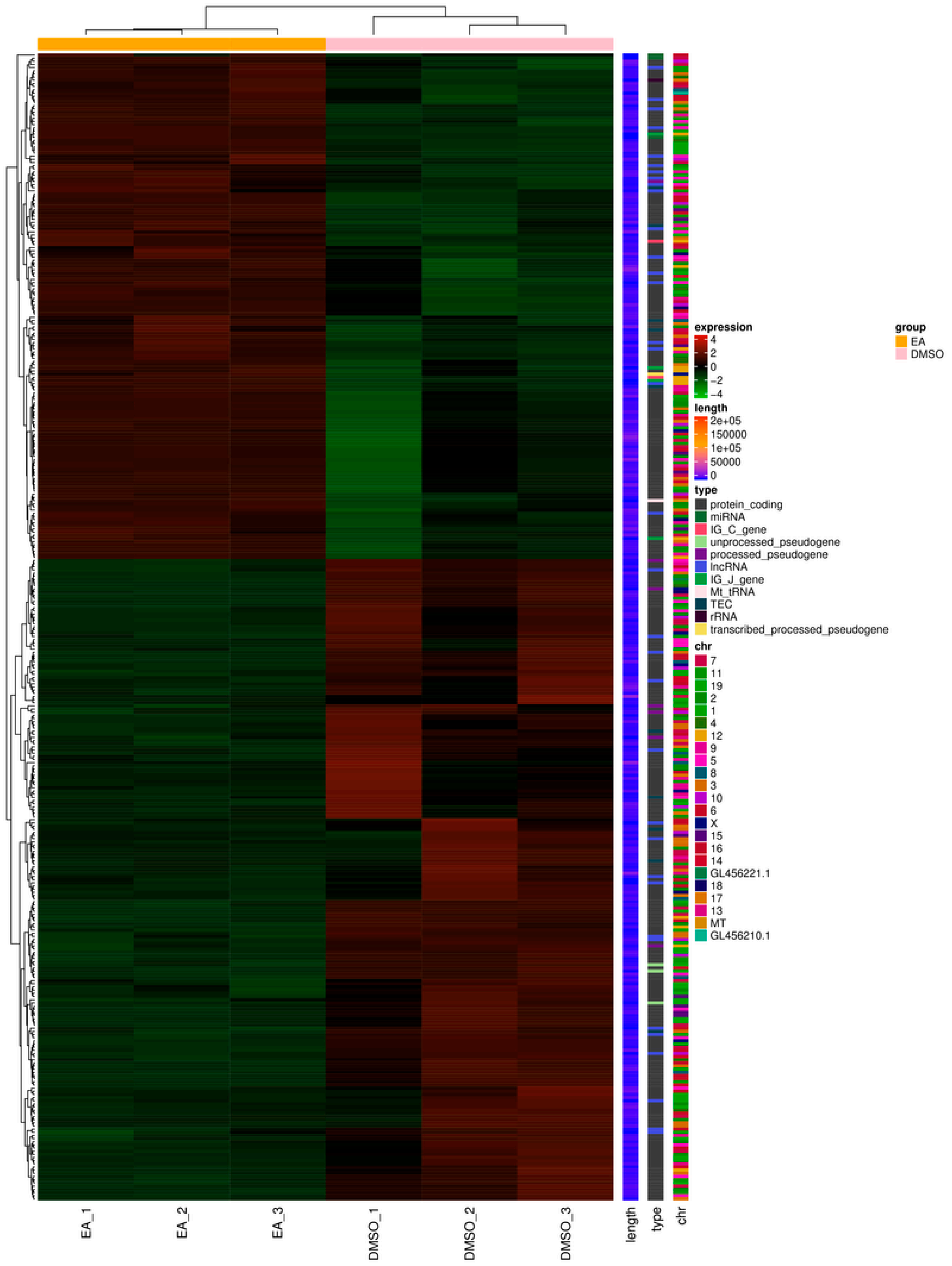

Figure. S3. Differential expression gene clustering heatmap of EA and DMSO treated groups.

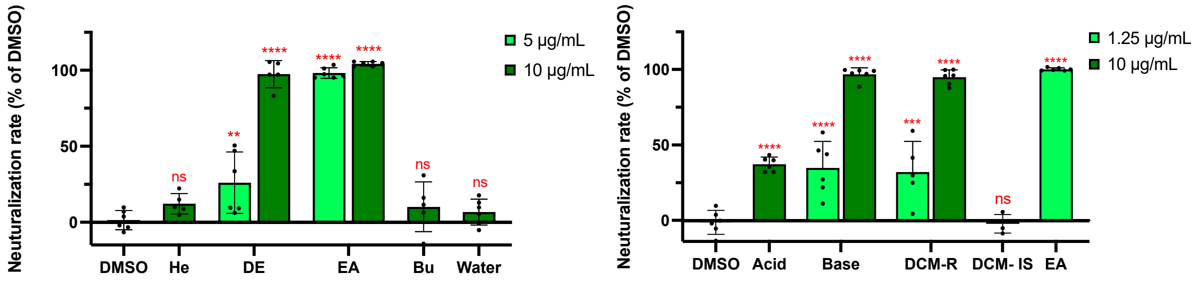

(A) (B)

Figure. S4. Screening results of subfractions extracted from papaya leaves juice (**A**) and from EA fraction (**B**) on HCoV-OC43 infected H1299 cells using in-cell ELISA. Data points and bar represent arithmetic shown are the mean ± SD of at least two independent tests performed. $^{*}P < 0.05$, $^{**}P < 0.01$, $^{***}P < 0.001$, $^{****}P < 0.0001$ as compared to DMSO group.

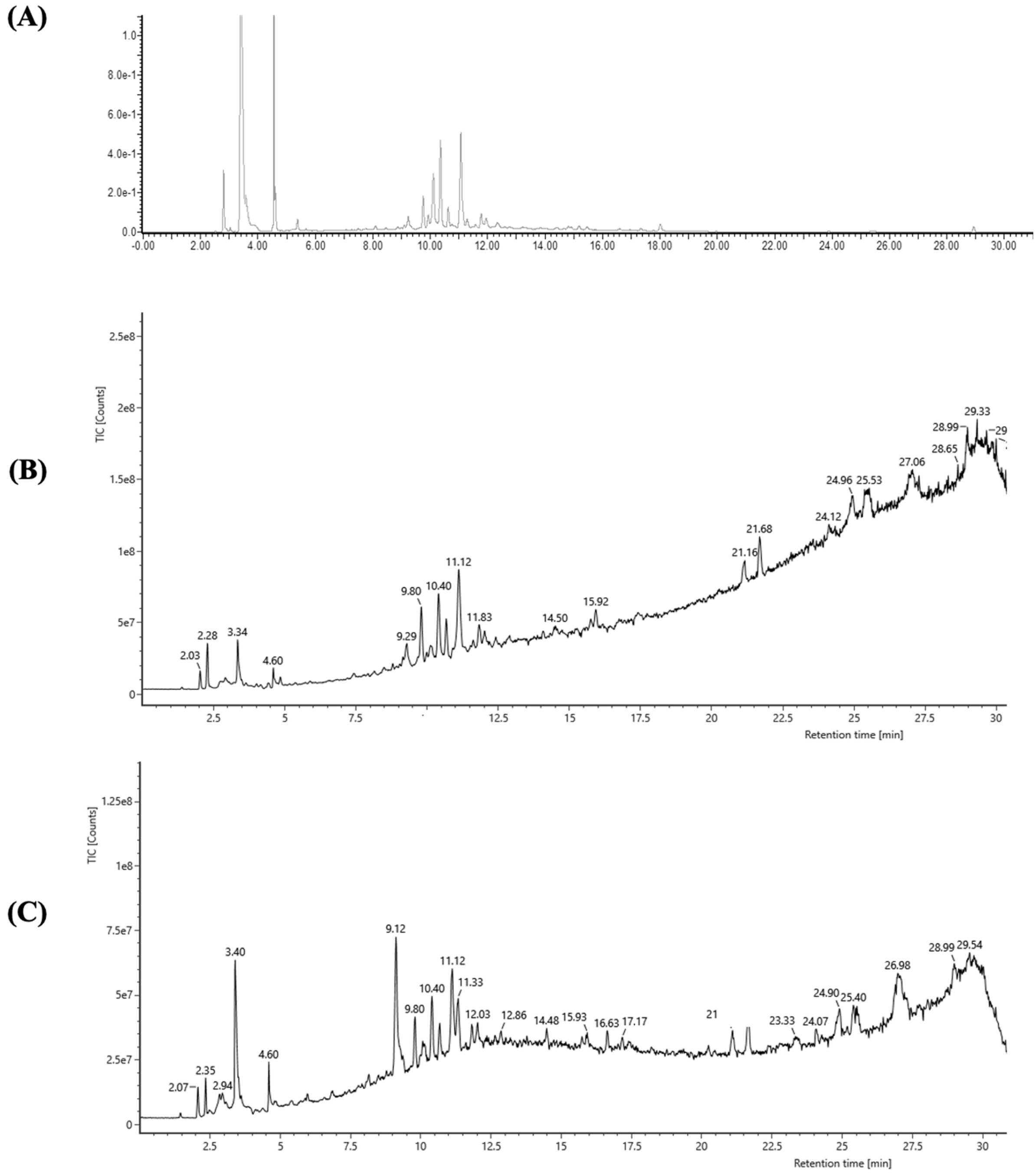

Figure. S5. HPLC profile (**A**) and total ion chromatogram at negative mode (**B**) and positive mode (**C**) of EA fraction.

(A)

(B)

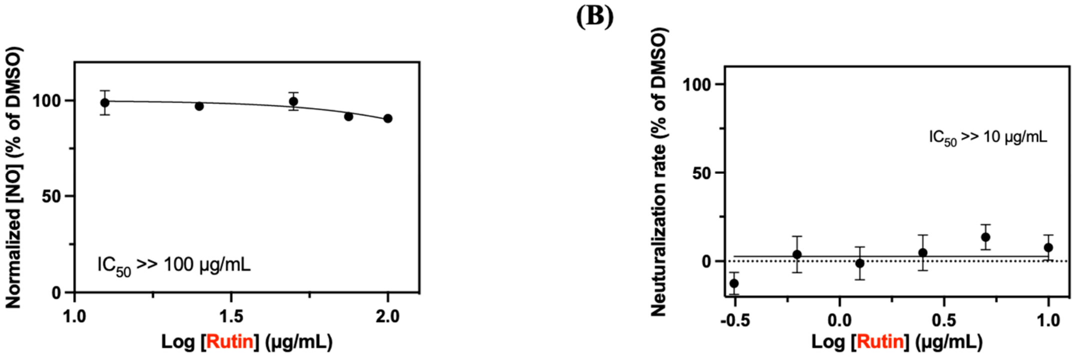

Figure. S6. Inhibitory effects of rutin on NO production in LPS-induced RAW 264.7 cells (**A**) and its function in neutralizing HCoV-OC43 on infected H1299 cells (**B**). Data points and bar represent arithmetic shown are the mean ± SD of at least two independent tests performed.
